# Association between Sudden Sensorineural Hearing Loss and Preexisting Thyroid Diseases: A Nationwide Case-Control Study in Taiwan

**DOI:** 10.3390/ijerph17030834

**Published:** 2020-01-29

**Authors:** Yao-Te Tsai, I-Jen Chang, Cheng-Ming Hsu, Yao-Hsu Yang, Chia-Yen Liu, Ming-Shao Tsai, Geng-He Chang, Yi-Chan Lee, Ethan I. Huang, Meng-Hung Lin, Chih-Wei Luan

**Affiliations:** 1 Department of Otorhinolaryngology-Head and Neck Surgery, Chang Gung Memorial Hospital, Chiayi 61363, Taiwan; yaote1215@gmail.com (Y.-T.T.); scm00031@gmail.com (C.-M.H.); b87401061@gmail.com (M.-S.T.); genghechang@gmail.com (G.-H.C.); ehuang@cgmh.org.tw (E.I.H.); 2 Department of Family Medicine, Chang Gung Memorial Hospital, Chiayi 61363, Taiwan; drchangijen@gmail.com; 3 Health Information and Epidemiology Laboratory, Chang Gung Memorial Hospital, Chiayi 61363, Taiwan; r95841012@ntu.edu.tw (Y.-H.Y.); qchiayen@gmail.com (C.-Y.L.); mattlin@cgmh.org.tw (M.-H.L.); 4 Department of Traditional Chinese Medicine, Chang Gung Memorial Hospital, Chiayi 61363, Taiwan; 5 Department of Otorhinolaryngology-Head and Neck Surgery, Chang Gung Memorial Hospital, Keelung 20445, Taiwan; Alberttylee@gmail.com; 6 Department of Otorhinolaryngology-Head and Neck Surgery, Chiayi Christian Hospital, Chiayi 61363, Taiwan

**Keywords:** thyroid diseases, hypothyroidism, hyperthyroidism, hearing impairment, sudden sensorineural hearing loss

## Abstract

*Background*: Little evidence is available about the risk of sudden sensorineural hearing loss (SSNHL) in patients with thyroid diseases. We assessed whether a diagnosis of thyroid disease, particularly hyperthyroidism or hypothyroidism, is associated with SSNHL risk in an Asian population. *Material and Methods:* This case-control study was conducted with population-based data from Taiwan’s National Health Insurance Research Database from January 2000 to December 2013. The case group comprised 3331 adult patients with newly diagnosed SSNHL, and four controls without SSNHL for each case matched by sex, age, monthly income, and urbanization level of residence. Underlying Thyroid diseases were retrospectively evaluated in the case and control groups. Multivariate logistic regression analyses were used to explore relations between thyroid diseases and SSNHL. *Results:* Of the 3331 cases, 5.7% had preexisting thyroid diseases, whereas only 4.0% of the 13,324 controls had the same condition. After adjustment for sex, age, monthly income, urbanization level of residence, history of hypertension, diabetes mellitus, chronic otitis media, and hyperlipidemia, associations were identified between a history of either hypothyroidism (adjusted odds ratio [AOR], 1.54; 95% CI, 1.02–2.32; *p* = 0.042) or hyperthyroidism (AOR, 1.41; 95% CI, 1.07–1.85; *p* = 0.015) and an elevated risk of SSNHL. In subgroup analysis, the correlation between hypothyroidism and increased SSNHL risk remained significant only for patients aged over 50 years (AOR, 1.61; 95% CI, 1.01–2.57; *p* = 0.045), and that between hyperthyroidism and SSNHL was significant only for female patients (AOR, 1.48; 95% CI, 1.09–2.01; *p* = 0.012). Treatment for hypothyroidism and hyperthyroidism did not alter the association in subgroup analyses. *Conclusion:* Preexisting hypothyroidism and hyperthyroidism appear associated with SSNHL susceptibility in Taiwan. Physicians should be wary of this elevated risk of SSNHL among patients with previously diagnosed thyroid dysfunction, especially women and patients aged more than 50 years.

## 1. Introduction

Sudden sensorineural hearing loss (SSHL) is usually defined as acute loss of more than 30 dB in three contiguous frequencies in less than 72 hours, and the incidence was reported to be 7.79 and 8.85 per 100,000 women and men, respectively [1]. Despite substantial improvements in neurotology, SSNHL etiology remains debated. Numerous etiologies, including infection, otologic disease, and trauma, have been confirmed but most cases are idiopathic [2]. SSNHL may be associated with a variety of underlying comorbidities such as iron deficiency anemia, chronic kidney disease, systemic lupus erythematosus, diabetes mellitus (DM), human immunodeficiency virus, psoriasis, osteoporosis, and chronic otitis media (COM) [3,4,5,6,7,8,9,10].

Thyroid diseases, which are globally prevalent endocrine disorders, may present with various manifestations. Studies have revealed that abnormal changes in serum thyroid hormone levels, such as hyperthyroidism and hypothyroidism, can cause both sensorineural and conductive hearing loss [11,12]. Moreover, a cross-sectional study indicated that thyroid diseases are more frequent in patients with SSNHL than in the general population, but information on the types of thyroid diseases are unavailable [13]. Expanding on these research findings, it is reasonable to hypothesize an association between the thyroid diseases and SSNHL. Nevertheless, a lack of relevant evidence prevents the formulation of salutary advices with regard to clinical practice. To gain a further understanding of SSNHL risk in patients with thyroid diseases, particularly hyperthyroidism and hypothyroidism, we used data from a nationwide claims database to elucidate our hypothesis. Significant correlations may indicate early risk factors for SSNHL.

## 2. Materials and Methods

### 2.1. Data Source and Study Design

We designed a case-control study using the Taiwanese National Health Insurance Research Database (NHIRD). The National Health Insurance (NHI) program was implemented by the Taiwan government in 1995 and covers over 99% of Taiwan’s residents [14]. The NHI created the NHIRD, comprising detailed information on prescriptions, surgical procedures, and clinic visits, as well as diagnostic codes in accordance with International Classification of Diseases, Ninth Revision, Clinical Modification (ICD-9-CM). Its nearly perfect coverage provides data suitable for nationwide population-based studies. The present study used data stored in the Longitudinal Health Insurance Database 2005 (LHID 2005), a representative NHIRD subdatabase containing medical claims made by 1 million NHI enrollees selected at random from 2005 through systematic sampling. The Taiwan National Health Research Institutes report that the health-care costs, age, and sex of individuals in the LHID2005 are not significantly different from those of the NHIRD [15]. The Institutional Review Board of Chang Gung Memorial Hospital approved of this study (201901124B1). Because all personal information in the database used is encrypted to ensure privacy, the board waived the obligation of obtaining informed consent.

### 2.2. Patient Selection and Case-Control Matching

**Figure 1:**
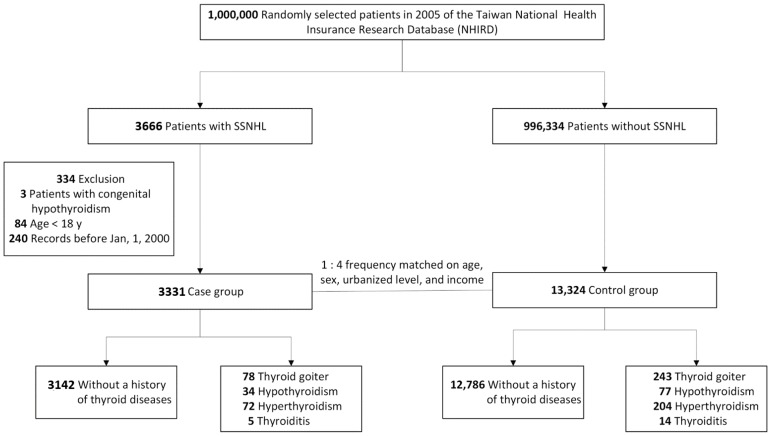
Flow diagram of participants selection and study design. SSNHL—sudden sensorineural hearing loss.

Figure 1 presents a flowchart of patient selection. We selected patients with a new SSNHL diagnosis in an inpatient claim or at fewest three outpatient claims between 1 January 2000, and 31 December 2013, for the case group. ICD-9-CM code 388.2 was used to identify SSNHL diagnoses. To enhance the accuracy of diagnosis, we only included patients with SSNHL coded by otolaryngologists. Because we were interested in the relationship between thyroid disease and SSNHL in adults, we excluded patients aged less than 18 years and those with congenital hypothyroidism, and selected 3331 patients with SSNHL for the case group. For high statistical power, we matched each case with four randomly selected controls without SSNHL from the LHID2005 for the case index date; the control group was frequency-matched by age, sex, urbanization level of residence, and monthly income. Thus, in total, we selected 13,324 controls.

### 2.3. Thyroid Disorders and Other Adjustments

The primary outcome we were interested in was the prevalence of preexisting thyroid diseases. To identify cases and controls with diagnoses of thyroid disease, we searched for *ICD-9-CM* codes 243 and 244 for hypothyroidism, 242 for hyperthyroidism, 240.xx to 241.xx for thyroid goiter, and 245.xx for thyroiditis. Patients were considered to have a thyroid disease if the diagnostic code appeared three times or more in outpatient visits or more than once in an inpatient setting. We established whether each individual had thyroid diseases before diagnosis of SSNHL in the case group and before the matched index date in the control group. We adjusted for sex, age, monthly income, urbanization level of residence, and the diagnoses of hypertension (HTN, *ICD-9-CM* codes 401-405), diabetes mellitus (DM, *ICD-9-CM* codes 250.xx and A-code A-181), chronic otitis media (COM, *ICD-9-CM* codes 381.1-381.3 and 382.1-382.3), and hyperlipidemia (*ICD-9-CM* codes 272.0-272.4) in inpatient care claims or in three or more outpatient claims before the index date. We analyzed covariates as binomial variables. Medication treatment for thyroid dysfunction, including levothyroxine sodium for hypothyroidism and methimazole and propylthiouracil for hyperthyroidism, was defined as use of the medication between diagnosis of thyroid disease and the index date. Surgical treatment of thyroid diseases, including partial and total thyroidectomy, was defined as the procedure occurring between diagnosis of thyroid disease and the index date.

### 2.4. Statistical Analysis

Categorical variables are given as a percentage and frequency, whereas continuous variables are given as a mean and standard deviation. We assessed differences in the clinical characteristics of the groups with Pearson chi-square testing. We conducted conditional logistic regression while controlling for potential covariates to explore the associations between thyroid diseases (thyroid goiter, hypothyroidism, hyperthyroidism, and thyroiditis) and the risk of SSNHL. We applied the logistic regression model in subgroup analysis to detect any associations between the treatment of hypothyroidism or hyperthyroidism and risk of SSNHL, adjusted for sex, age, monthly income, urbanization level of residence, and covariates. Analysis was performed with SAS version 9.4 (SAS Institute, Cary, NC, USA), and statistical significance was indicated by a 2-sided *p* value of < 0.05.

## 3. Results

Between 1 January 2000, and 31 December 2013, a total of 3331 patients with newly coded SSNHL met the criteria for inclusion in the case group, and 13,324 patients without SSNHL were matched as controls, among whom majorities were male (53.6%) or aged over 50 years (61.2%). The mean age of the 16,655 study individuals was 53.1 years, with a standard deviation of 15.4 years. The case and control groups differed significantly in terms of the proportions with a record of thyroid diseases (*p* = 0.001), DM (*p* < 0.001), COM (*p* < 0.001), HTN (*p* < 0.001), or hyperlipidemia (*p* < 0.001). In the case group, 34 patients (1.0%) had preexisting hypothyroidism, and the control group included 77 such patients (0.6%, z-score = 2.575; *p* = 0.005). For hyperthyroidism, 72 cases (2.2%) and 204 (1.5%) controls had the condition (z-score = 2.297; *p* = 0.011, Table 1).

After adjustments for age, sex, urbanization level of residence, monthly income, and covariates, both hypothyroidism and hyperthyroidism were significantly associated with SSNHL (adjusted odds ratio [AOR], 1.54; 95% CI, 1.02–2.32; *p* = 0.042 and AOR, 1.41; 95% CI, 1.07–1.85; *p* = 0.015, respectively, Table 2). For patients with a history of thyroid goiter or thyroiditis, no significant difference was observed in the risk of SSNHL (AOR, 1.24; 95% CI, 0.96–1.61; *p* = 0.106, and AOR, 1.35; 95% CI, 0.48–3.78; *p* = 0.569, respectively). Stratification by age group revealed that the association between SSNHL and hypothyroidism was significant only among those aged 50 years and older (AOR, 1.61; 95% CI, 1.01–2.57; *p* = 0.045); this association disappeared in those aged 50 years or under. Besides, significant associations were not discovered among age subgroups for any other thyroid disease. Subgroup analysis stratified by sex revealed significantly increased odds ratio of SSNHL for female patients with hyperthyroidism (AOR, 1.48; 95% CI, 1.09–2.01; *p* = 0.012) but no significant relationships between thyroid diseases and increased SSNHL risk among men.

In the subgroup analysis (Table 3), we investigated whether prior medication or surgical treatment for hyperthyroidism or hypothyroidism would alter the risk of developing SSNHL. The analyses revealed no statistically significant differences between treatments for hypothyroidism or hyperthyroidism and the risk of developing SSNHL (AOR, 0.61; 95% CI, 0.21–1.76; *p* = 0.358 and AOR, 0.77; 95% CI, 0.43–1.38; *p* = 0.379, respectively).

## 4. Discussion

According to our review of literature, this population-based case-control study is the first to investigate the associations between SSNHL and thyroid diseases. Obtaining an adequately sized sample from a single medical institution is challenging because of the rarity of SSNHL. Through the use of a nationwide database, we could overcome this challenge and obtain an adequate sample of patients with SSNHL, with minimal selection bias. To minimize the effects of potential covariates, we used AOR to compare the groups. After adjustment for a variety of covariates, including DM, HTN, COM, and hyperlipidemia, an association of preexisting hyperthyroidism and hypothyroidism with an elevated risk of SSNHL was discovered in this case-control study involving 16,655 individuals. In subgroup analysis, the association between hypothyroidism and SSNHL was most prominent among patients older than 50 years; moreover, the association between hyperthyroidism and SSNHL appeared significant for women but not for men. Prior treatment for thyroid dysfunction was not significantly associated with reduced risk of SSNHL. This study extends the SSNHL literature but also increases awareness of the high SSNHL risk associated with hypothyroidism and hyperthyroidism, thus facilitating timely diagnosis and intervention.

Human and animal studies have shown that the development of auditory system relies on the presence of appropriate thyroid hormone levels [16].

Lautermann et al. investigated the distribution of the thyroid hormone receptor in the spiral ganglion and outer and inner cochlear hair cells of rats; immunohistochemistry provided evidence suggesting that thyroid hormone influences cochlear development [17]. Cordas et al. found through an in situ hybridization study that Thra and Thr*b* encode thyroid hormone receptors α1 and β in the tympanic membrane, immature ossicles, and middle ear mesenchyme of mice [18]. These findings imply that the middle and inner ear may be targets sensitive to changes in serum levels of thyroid hormone. Studies have indicated that hearing ability may be impaired in patients with endemic cretinism, hypothyroidism, or thyroid hormone resistance [19,20,21,22]. Hypothyroidism and hyperthyroidism may lead to conductive hearing impairment resulting from eustachian tube or middle ear mucosal edema, or to sensorineural hearing loss resulting from endocochlear, retrocochlear, or central hearing impairment [23]. Psaltakos et al. recorded otoacoustic emissions and performed tympanometry and pure-tone audiometry for 52 patients with thyroid carcinoma before and 6 to 8 weeks after total thyroidectomy; acute iatrogenic hypothyroidism elevated hearing thresholds and caused subclinical damage to cochlear function [24]. However, the ability of thyroxine treatment to improve hearing ability in patients with hypothyroidism remains uncertain because the interactions are complex between thyroid hormones and associated receptors in the middle and inner ear [22].

Only a few researchers have investigated the association between dysfunctional thyroid and SSNHL. Oiticica et al. conducted a study on 166 patients with SSNHL and observed that thyroid dysfunction was more than twice as common among these patients than among the general population [13]. Nakashima et al. explored SSNHL risk factors in a case-control study with 109 patients, reporting that patients with a history of thyroid disease had a higher odds ratio for SSNHL than those without such history [25]. However, they did not distinguish between types of thyroid disease when analyzing the associated risk of SSNHL; in clinical practice, this is a crucial distinction. Narozny et al. also suggested that hypothyroidism is associated with inferior hearing outcomes in SSNHL [26]. Here, we identified significant associations of preexisting hypothyroidism and hyperthyroidism with SSNHL risk. The reason for these associations may be that thyroid autoantibodies mediate peripheral or central hearing organ dysfunction, increasing susceptibility to SSNHL [27]. Moreover, studies have indicated that thyroid dysfunction is related to hypercoagulability and venous thrombosis, which may impair the cochlear circulation, thus causing SSNHL [28,29]. Impaired hearing ability has been reported in patients with autoimmune-related hyperthyroidism, particularly at high frequencies [30]. Both subclinical and overt hyperthyroidism modified the coagulation-fibrinolytic balance and induced a prothrombotic state [31,32], which may also have increased the risk of SSNHL [33,34]. The aforementioned results imply that preexisting thyroid diseases are involved in SSNHL pathogenesis. Large, prospective, randomized controlled trials are warranted to validate our results.

Sex subgroup analysis indicated no significant association between preexisting thyroid disease and SSNHL among men; this finding is consistent with those revealing that various thyroid diseases, including hypothyroidism and hyperthyroidism, are more common in women, and that the overall incidence of thyroid diseases increases more rapidly with age in women than in men [35,36,37]. Sex differences in the association between SSNHL and thyroid diseases may therefore be partly explained by the low incidence of thyroid diseases in men in the present study. Although the reason why thyroid diseases are disproportionately more prevalent among women is unknown, extrathyroidal iodine storage in breast and cervical tissue [38] and the regulation of thyroid function by estradiol are likely involved [39]. Among patients with thyroid dysfunction, those receiving medication or surgical treatment had a decreased odds ratio of SSNHL compared with those not undergoing treatment, but these results were not statistically significant. Whether treatment for thyroid disfunction can reduce the risk or severity of subsequent SSNHL should be explored using a large, prospective, cohort study in the future.

This study’s strengths include its large sample drawn from a national health care database, case-control design, and adjustment for SSNHL risk factors. However, this study also has several limitations. First, SSNHL diagnoses were determined by *ICD-9-CM* codes rather than audiological examination; therefore, our results might underestimate the incidence of SSNHL. However, such an underestimation would have affected both the case and control groups. Second, detailed audiometry data and thyroid hormone levels are not available in the claims database; thus, we could not test whether the severity of thyroid dysfunction affects the severity of SSNHL. Third, certain biases might influence our results because data regarding several potential SSNHL risk factors, such as prior occupational noise exposure, cigarette smoking, and alcohol consumption, are unavailable in the NHIRD [40]. Although our results reached statistical significance and extend the literature on the etiology of SSNHL, readers should consider the aforementioned limitations when interpreting our results.

## 5. Conclusions

Our nationwide case-control study suggests that a history of hypothyroidism or hyperthyroidism, particularly hypothyroidism in patients aged over 50 years and hyperthyroidism in female patients, is correlated with an elevated risk of SSNHL in Taiwan. We suggest that when thyroid dysfunction is diagnosed, a baseline audiogram is intended to provide a reference for future comparison. For timely diagnosis and treatment of SSNHL, clinicians should advise patients with these risk factors to notify a doctor should they experience acute changes in hearing function.

## Figures and Tables

**Table 1:** Demographic Characteristics of Sensorineural Hearing Loss and Control Groups.

Variables	SSNHL (*N* = 3331)		Non-SSNHL (N = 13,324)		*p*-Value ^a^
	*n*	%	n	%	
**Sex**					1.000
Men	1786	53.6	7144	53.6	
Women	1545	46.4	6180	46.4	
**Age (years)**					1.000
18–49	1294	38.9	5176	38.9	
≥50	2037	61.2	8148	61.2	
**Monthly income (NTD)** 0	856	25.7	3424	25.7	1.000
1–15840	577	17.3	2308	17.3	
15841–25000	988	29.7	3952	29.7	
≥25001	910	27.3	3640	27.3	
**Urbanization level**					1.000
1(City)	920	27.6	3680	27.6	
2	1595	47.9	6380	47.9	
3	573	17.2	2292	17.2	
4 (Village)	243	7.3	972	7.3	
**Thyroid diseases**					0.001
Thyroid goiter	78	2.3	243	1.8	
Hypothyroidism	34	1.0	77	0.6	
Hyperthyroidism	72	2.2	204	1.5	
Thyroiditis	5	0.2	14	0.1	
None	3142	94.3	12786	96.0	
**Age < 50 y/o**					0.074
Thyroid goiter	19	1.5	59	1.1	
Hypothyroidism	7	0.5	19	0.4	
Hyperthyroidism	26	2.0	67	1.3	
Thyroiditis	3	0.2	4	0.1	
None	1239	95.8	5027	97.1	
**Age ≥ 50 y/o**					0.008
Thyroid goiter	59	2.9	184	2.3	
Hypothyroidism	27	1.3	58	0.7	
Hyperthyroidism	46	2.3	137	1.7	
Thyroiditis	2	0.1	10	0.1	
None	1903	93.4	7759	95.2	
**Female**					0.003
Thyroid goiter	58	3.8	192	3.1	
Hypothyroidism	25	1.6	58	0.9	
Hyperthyroidism	60	3.9	162	2.6	
Thyroiditis	1	0.1	14	0.2	
None	1401	90.7	5754	93.1	
**Male**					0.001
Thyroid goiter	20	1.1	51	0.7	
Hypothyroidism	9	0.5	19	0.3	
Hyperthyroidism	12	0.7	42	0.6	
Thyroiditis	4	0.2	0	0	
None	1741	97.5	7032	98.4	
**Medication for thyroid dysfunction**	135	4.1	427	3.2	0.015
**Thyroidectomy**	12	0.4	51	0.4	0.850
**Covariates**					
History of DM	650	19.5	1713	12.9	<0.001
History of COM	62	1.9	98	0.7	<0.001
History of HTN	1175	35.3	3879	29.1	<0.001
History of hyerlipidemia	786	23.6	2340	17.6	<0.001

Abbreviations: SSNHL, sudden sensorineural hearing loss; NTD, New Taiwan dollar; DM, diabetes mellitus; COM, chronic otitis media; and HTN, hypertension. ^a^ *p* values calculated with Pearson chi-square test.

**Table 2:** Adjusted Odds Ratios of Sensorineural Hearing Loss Associated With Various Thyroid Diseases.

Variables	Adjusted OR ^a^ (95% CI)	*p*-Value
**Overall**		
Without thyroid disorders	1 [Reference]	NA
Thyroid goiter	1.24 (0.96−1.61)	0.106
Hypothyroidism	1.54 (1.02−2.32)	0.042
Hyperthyroidism	1.41 (1.07−1.85)	0.015
Thyroiditis	1.35 (0.48−3.78)	0.569
**Age < 50 y/o**		
Without thyroid disorders	1 [Reference]	NA
Thyroid goiter	1.22 (0.72−2.07)	0.462
Hypothyroidism	1.40 (0.57−3.39)	0.462
Hyperthyroidism	1.41 (0.88−2.26)	0.151
Thyroiditis	3.06 (0.68−13.74)	0.145
**Age ≥ 50 y/o**		
Without thyroid disorders	1 [Reference]	NA
Thyroid goiter	1.25 (0.92−1.68)	0.153
Hypothyroidism	1.61 (1.01−2.57)	0.045
Hyperthyroidism	1.36 (0.97−1.92)	0.075
Thyroiditis	0.73 (0.16−3.38)	0.690
**Women**		
Without thyroid disorders	1 [Reference]	NA
Thyroid goiter	1.17 (0.86−1.58)	0.320
Hypothyroidism	1.46 (0.90−2.37)	0.125
Hyperthyroidism	1.48 (1.09−2.01)	0.012
Thyroiditis	0.28 (0.04−2.12)	0.217
**Men**		
Without thyroid disorders	1 [Reference]	NA
Thyroid goiter	1.54 (0.91−2.61)	0.106
Hypothyroidism	1.73 (0.77−3.85)	0.183
Hyperthyroidism	1.14 (0.60−2.18)	0.688
Thyroiditis	NA	NA
DM	1.47 (1.31−1.64)	<0.001
COM	2.50 (1.81−3.45)	<0.001
HTN	1.21 (1.09−1.33)	<0.001
Hyerlipidemia	1.21 (1.09−1.35)	<0.001

Abbreviations: OR, odds ratio; NA, not applicable; DM, diabetes mellitus; COM, chronic otitis media; HTN, hypertension; ^a^ odds ratio adjusted for sex, age, urbanization level, monthly income, and covariates.

**Table 3:** Subgroup Analysis for Treatment-Adjusted Odds Ratio of Sensorineural Hearing Loss Associated With Thyroid Diseases.

Variable	Adjusted OR ^a^ (95% CI)	*p*-Value
**Participants with hypothyroidism**		
Without TD medications	1 [Reference]	NA
With TD medications ^b^	0.61 (0.21–1.76)	0.358
**Participants with hyperthyroidism**		
Without TD medications and thyroidectomy	1 [Reference]	NA
With TD medications ^c^ or thyroidectomy ^d^	0.77 (0.43–1.38)	0.379

Abbreviations: TD, thyroid disease; NA, not applicable; OR, odds ratio; ^a^ odds ratio adjusted for sex, age, urbanization level, monthly income, and covariates; ^b^ hypothyroidism medication: levothyroxine sodium; ^c^ hyperthyroidism medications: methimazole, propylthiouracil; and ^d^ partial or total thyroidectomy.
